# Unusual acral bullous dyshidrosiform rash associated with acute human herpes virus-6 infection in a 2-year-old girl

**DOI:** 10.1016/j.jdcr.2025.09.003

**Published:** 2025-09-19

**Authors:** Alessandro Svizzero, Andrea Michelerio, Marco Nisi, Arber Selimi, Valeria Brazzelli

**Affiliations:** a Institute of Dermatology, Fondazione IRCCS Policlinico San Matteo, Pavia, Italy; b Department of Clinical, Surgical, Diagnostic and Pediatric Sciences, Università degli Studi di Pavia, Pavia, Italy

**Keywords:** acral dyshidrosiform rash, child, HHV6

## Introduction

Human herpesvirus 6 (HHV-6) is a common virus that primarily affects young children. It typically causes roseola infantum, which is characterized by a high fever followed by a transient maculopapular rash. Rarely, HHV-6 infection can lead to atypical dermatologic manifestations.^1^ Here, we present the case of a two-year-old girl who developed a dyshidrosiform rash on her hands after experiencing an acute HHV-6 infection.

## Case report

**Fig 1:**
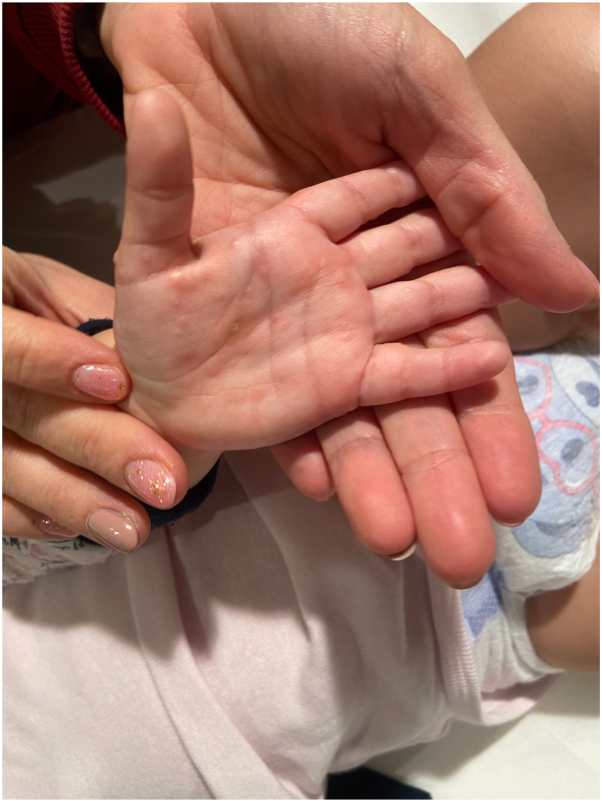
Vesicles and small bullae with dyshidrosiform appearance on the hands.

**Fig 2:**
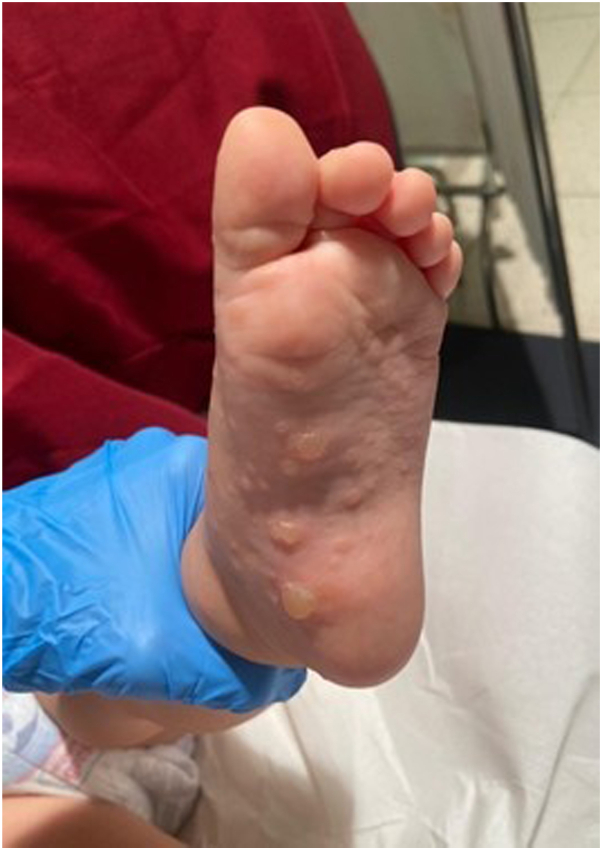
Tense bullae on the plantar region.

A 2-year-old girl presented to our dermatology clinic with a bullous rash on her hands (Fig 1) and feet (Fig 2). The rash was characterized by tense vesiculobullous lesions without an erythematous base, which is consistent with a dyshidrosiform pattern.^2^ The patient's mother reported that the rash had appeared approximately 1 week after the child had experienced a high fever lasting 5 days and irritability.

The patient had no significant past medical history and was previously healthy. There was no family history of immunodeficiency or dermatological conditions.

Blood tests were performed to further investigate the cause of the rash. Routine laboratory tests and inflammatory markers showed no significant abnormalities. Additionally, anti-desmoglein (anti-Dsg1 and anti-Dsg3) antibodies, anti-bullous pemphigoid (anti-BP180 and anti-BP230) antibodies, and anti-collagen VII antibodies were negative. Anti-tissue transglutaminase 3 autoantibodies were also negative. Microbiological investigations included testing for IgG and IgM antibodies to Mycoplasma pneumoniae, Epstein-Barr virus, cytomegalovirus, parvovirus B19, and coxsackievirus. Notably, the patient had cytomegalovirus IgG at 118 U/mL (positive if >14 U/mL) and Parvovirus B19 IgG at 42.20 U/mL (positive if >2.5 U/mL) but had negative IgM. Calibrated quantitative real-time polymerase chain reaction to evaluate the presence and copy number of HHV-6 and HHV-7 revealed the presence of more than 40,000 copies/mL of HHV-6 DNA, indicating an active infection.^3^

The child was treated with betamethasone 0.1% cream applied twice daily and emollients for 10 days until the skin symptoms resolved. Four weeks later, HHV-6 DNA levels were undetectable, confirming resolution of the infection.

## Discussion

HHV-6 is known to cause roseola infantum, a common childhood illness characterized by a high fever followed by a rash.^4^ However, the development of a dyshidrosiform rash is atypical. Acute HHV-6 or HHV-7 infections can present with atypical manifestations, including acral involvement, as documented in existing literature in adults.^5^ The pathogenesis of this palmoplantar vesiculobullous eruption is unclear; however, some observations support a virus-mediated mechanism. Transcriptomic profiling of vesicular hand eczema shows marked upregulation of innate host-defense genes and downregulation of epidermal differentiation markers. This suggests that exaggerated innate immunity can promote intraepidermal vesiculation.^6^ In this context, HHV-6 infection may act as a trigger by directly infecting epithelial and immune cells or by promoting local immune dysregulation. HHV-6 exhibits broad cellular tropism, including keratinocytes, dendritic cells, fibroblasts, and endothelial cells, which supports its potential for direct cutaneous involvement. Additionally, the virus modulates host immunity by inducing proinflammatory cytokines (eg, interleukin-1β, tumor necrosis factor-α, and interferon-α) and expressing proteins (eg, U21 and U24) that interfere with antigen presentation and T cell signaling.^3^ These combined effects, direct epithelial involvement and immune-mediated inflammation, may lead to vesiculation, even in immunocompetent children. The relative immaturity of the epidermal barrier in early childhood may further amplify these mechanisms, predisposing individuals to bullous changes.

## Conclusion

This case underscores the importance of considering viral etiologies, such as HHV-6, when making a differential diagnosis of vesiculobullous eruptions in children. Atypical viral presentations can resemble primary immunobullous or eczematous disorders, making accurate virologic investigation crucial for proper diagnosis and management. This observation contributes to the growing body of evidence indicating that HHV-6 and HHV-7 may be associated with a wider range of cutaneous manifestations, including those in immunocompetent pediatric patients. Recognizing these patterns may facilitate timely diagnosis and appropriate, conservative treatment.

## Conflicts of interest

None disclosed.
